# Elbow Joint Angles in Elbow Flexor Unilateral Resistance Exercise Training Determine Its Effects on Muscle Strength and Thickness of Trained and Non-trained Arms

**DOI:** 10.3389/fphys.2021.734509

**Published:** 2021-09-16

**Authors:** Shigeru Sato, Riku Yoshida, Ryosuke Kiyono, Kaoru Yahata, Koki Yasaka, João Pedro Nunes, Kazunori Nosaka, Masatoshi Nakamura

**Affiliations:** ^1^Institute for Human Movement and Medical Sciences, Niigata University of Health and Welfare, Niigata, Japan; ^2^Department of Physical Therapy, Niigata University of Health and Welfare, Niigata, Japan; ^3^Metabolism, Nutrition, and Exercise Laboratory, Physical Education and Sport Center, Londrina State University, Londrina, Brazil; ^4^Centre for Exercise and Sports Science Research, School of Medical and Health Sciences, Edith Cowan University, Joondalup, WA, Australia

**Keywords:** maximal voluntary contraction torque, isometric, concentric, eccentric, cross-education effect, range of motion

## Abstract

The present study compared two unilateral arm curl resistance exercise protocols with a different starting and finishing elbow joint angle in the same ROM for changes in elbow flexors strength and muscle thickness of the trained and non-trained arms. Thirty-two non-resistance trained young adults were randomly assigned to one of the three groups: extended joint training (0°–50°; EXT, *n* = 12); flexed joint training (80°–130°; FLE, *n* = 12); and non-training control (*n* = 8). The exercise training was performed by the dominant arms twice a week for 5 weeks with gradual increases in the training volume over 10 training sessions, and the non-dominant (non-trained) arms were investigated for the cross-education effect. Maximal voluntary contraction torque of isometric (MVC-ISO), concentric (MVC-CON), and eccentric contractions (MVC-ECC), and thickness (MT) of biceps brachii and brachialis of the trained and non-trained arms were assessed at baseline and 4–8 days after the last training session. The control group did not show significant changes in any variables. Significant (*P* < 0.05) increases in MVC-ISO torque (16.2 ± 12.6%), MVC-CON torque (21.1 ± 24.4%), and MVC-ECC torque (19.6 ± 17.5%) of the trained arm were observed for the EXT group only. The magnitude of the increase in MT of the trained arm was greater (*P* < 0.05) for EXT (8.9 ± 3.9%) than FLE (3.4 ± 2.7%). The cross-education effect was evident for MVC-ISO (15.9 ± 14.8%) and MVC-CON (16.7 ± 20.0%) torque of the EXT group only. These results suggest that resistance training at the extended elbow joint induces greater muscle adaptations and cross-education effects than that at flexed elbow joint.

## Introduction

In resistance training, range of motion (ROM) is a factor influencing its effects on muscle adaptations (Schoenfeld and Grgic, 2020). Schoenfeld and Grgic (2020) showed in their recent systematic review article that performing resistance training through a full ROM induced greater effects on hypertrophy of the lower body musculature when compared with training with a partial ROM. They also stated that research on the effects of ROM on upper limb muscles was limited and conflicting. To the best of our knowledge, only two studies have examined the effects of ROM in upper limb muscle resistance training on muscle strength and muscle hypertrophy.

Pinto et al. (2012) reported that the increase in elbow flexion one-repetition maximum (1-RM) strength was significantly greater after a full ROM (0°–130° elbow flexion) protocol (26%) than a partial ROM (50°–100° elbow flexion) protocol (16%), but the increase in brachialis plus biceps thickness was similar between the full (10%) and partial ROM (8%) groups. Goto et al. (2019) compared a full ROM (0°–120° elbow flexion) and a partial ROM (45°–90° elbow flexion) triceps extension resistance training for changes in muscle strength and muscle cross-sectional area (CSA) of the elbow extensors performed by resistance-trained men three times a week for 8 weeks. They showed that the partial ROM group had significantly greater increases in maximum voluntary contraction (MVC) torque (40%) and CSA (49%) than the full ROM group (24, 28%). They speculated that the resistance training in partial than full ROM would induce greater intramuscular hypoxia, which would increase muscle protein synthesis, growth hormone, and mammalian targets of rapamycin (mTOR) signaling, inducing greater muscle adaptations. When a full and a partial ROM resistance exercise protocol are compared, not only the ROM *per se* but also muscle length in the ROM appears to affect its training effects. Thus, the effects of muscle length in a function of joint angle should be considered for resistance training protocols.

In relation to the muscle length effect, Noorkõiv et al. (2015) compared isometric resistance training of the knee extensors at a longer muscle length (75°–100° knee flexion) vs. a shorter muscle length (30°–50° knee flexion), performed 5 times of 5 s per session, 3 times a week for 6 weeks. They showed that maximal voluntary concentric contraction torque (12–13%) and knee extensor muscle volume (4.8–8.2%) increased significantly only after the longer muscle length training. In a dynamic resistance exercise study, Nunes et al. (2020) compared two types of bicep curl training with a cable-pulley system which induced greater torque at short muscle lengths vs. a barbell which could apply greater torque at long muscle lengths, performed three times a week for 10 weeks for changes in muscle strength and thickness of the elbow flexors. They showed that maximum voluntary isometric contraction (MVC-ISO) strength at a long muscle length (20° elbow flexion) increased significantly greater after the barbell (39%) than the cable-pulley training (30%) without a difference in biceps brachii thickness increase. Nosaka et al. (2005) compared two protocols of eccentric exercise of the elbow flexors with different starting positions of the elbow joint, 50°–0° elbow flexion (long muscle lengths) and 130°–80° elbow flexion (short muscle lengths), and showed greater muscle damage indicated by decreases in MVC-ISO strength and increases in muscle soreness, upper arm circumference, plasma creatine kinase and magnetic resonance image (MRI) T2 relaxation time after the long than short muscle length protocol. These studies suggest that dynamic resistance training effects are different between long and short muscle length protocols with the same range of motion. However, no previous study has investigated the effects of elbow joint ROM in which muscles are activated in different lengths (i.e., long vs. short muscle lengths) on muscle strength changes and muscle hypertrophy.

It has been reported that unilateral resistance training increases muscle strength in the contralateral non-trained homologous muscle, which is referred to as the cross-education effect (Manca et al., 2017, 2021). Manca et al. (2021) have concluded that the main mechanisms underpinning the cross-education effect are neural adaptations; particularly changes in cortical excitability in the primary motor and supplementary motor areas ipsilateral to the trained limb. A meta-analysis by Manca et al. (2017) showed that there was a positive correlation between the degree of muscle strength increase in the trained limb and that of the non-trained limb. Carr et al. (2021) have recently reported a greater reduction in maximal force due to fatigue after intermittent maximal isometric contractions at a long than a short muscle length in biceps brachii. In addition, Fariñas et al. (2019) mentioned that the set configuration causing more fatigue showed a greater adaptation of the central nervous system, which showed a greater cross-education effect in the non-trained arm. If the resistance training effects are affected by the muscle lengths, it may be that the cross-education effect is also different between the long and short muscle length protocols. However, this does not appear to have been investigated in the previous studies.

Therefore, the purpose of this study was to compare two unilateral arm curl protocols in which the ROM was the same, but the starting and finishing elbow joint angles were different; 0°–50° (more extended joint exercise, presumably long muscle lengths) and 80°–130° (more flexed joint exercise, presumably short muscle lengths) for changes in elbow flexors strength and biceps brachii and brachialis muscle thickness of the trained and non-trained arms following a short (5-week) training performed twice a week by untrained young adults. A previous study (Sato et al. [in press]) reported that the 5-week training with gradual increases in the training volume over 10 sessions increased muscle strength (22.5%) and muscle thickness (7.1%) of the elbow flexors. It was hypothesized that the magnitude of increases in the strength and muscle thickness would be greater for the extended than flexed joint protocol for both trained and non-trained arms.

## Materials and Methods

### Study Design

A randomized repeated measures experimental design was used to compare the effects of unilateral elbow flexor resistance training at more extended joint angles (elbow joint angle: 0°–50°, EXT) and that at more flexed joint angles (80°–130°, FLE) on muscle strength and muscle thickness. The dependent variables consisted of maximum voluntary isometric contraction (MVC-ISO) torque at four different angles (10°, 50°, 90°, 130° elbow flexion), maximum voluntary concentric contraction (MVC-CON) torque at two angular velocities (60°/s, 180°/s), maximum voluntary eccentric contraction (MVC-ECC) torque at 60°/s and muscle thickness (MT) of biceps brachii and brachialis of the trained and untrained arms. In both EXT and FLE groups, the dominant arm was trained, and the non-trained arm was used to investigate the cross-education effect. A control group that did not perform the training was also included to examine changes in the variables over 5 weeks.

A familiarization session was set 1 week prior to the baseline measurements, and all participants practiced the MVC-ISO, MVC-CON, and MVC-ECC torque measurements on both trained (dominant) and non-trained (non-dominant) arms. The resistance training was performed twice a week with a rest period of at least 48 h between sessions for 5 weeks (Sato et al., 2021). All participants were instructed to refrain from any systematic training outside the study for the experimental period.

### Participants

A total of 32 (19 male and 13 female) healthy university students who were free from any orthopedic disorders of the upper extremity, had no history of previous neuromuscular or chronic diseases, and had not performed a structured resistance training in the past 6 months, participated in the present study. They were allocated to the one of three groups randomly as follow; EXT group (*n* = 12; 7 male, 5 female; age: 20.7 ± 0.9 year; height: 167.1 ± 9.0 cm; body mass: 60.9 ± 11.4 kg), FLE group (*n* = 12; 8 male, 4 female; age: 21.4 ± 1.4 year; height: 165.7 ± 7.5 cm; body mass: 58.8 ± 9.9 kg), or control group (*n* = 8; 4 male, 4 female; age: 21.1 ± 0.6 year; height: 164.3 ± 6.6 cm; body mass: 57.2 ± 7.9 kg). There were no significant differences in age, height, and body mass among groups.

The effect size was estimated from the study by Pinto et al. (2012), who reported the effect size of 0.81 for the difference in muscle strength increase between a partial and a full ROM resistance training protocol of the elbow flexors. With a power of 0.95 and an α of 0.05, the sample size was estimated that at least 8 subjects were necessary for each group. Considering the estimation error, 12 subjects were recruited for the training groups, and 8 subjects were recruited for the control group. Among the 32 participants, all except one in the FLE group were right-hand dominant based on the Edinburgh Handedness Inventory (Oldfield, 1971). All participants were briefed on the study purpose and procedures, and a written consent was obtained from each participant. This study was approved by the Ethics Committee of Niigata University of Health and Welfare. The study was conducted in conformity with the policy statement regarding the use of human subjects by the Declaration of Helsinki.

### Training Protocol

The resistance training was performed by the dominant arm. Training intensity was increased progressively from 30% (1st session) to 50% (2nd and 3rd sessions), 70% (4th and 5th sessions), 80% (6th and 7th sessions), 90% (8th and 9th sessions), and 100% (10th session) of the MVC-ISO torque at 50° for the EXT, and at 90° for the FLE group measured before the training. The dominant arm of each participant was positioned on a preacher curl bench in a seated position, with 45° shoulder flexion and forearm supination (Nunes et al., 2020). Each session consisted of 3 sets of 10 repetitions (30 repetitions in total). The contraction tempo was indicated by a metronome, and each subject was instructed to move a dumbbell for the concentric phase and the eccentric phase in 2 s each. The range of motion was from 0° to 50° in the EXT group and from 80° to 130° in the FLE group (Nosaka et al., 2005). If a participant had difficulty controlling the dumbbell movement during training at a high intensity (80–100% MVC-ISO torque), the investigator assisted the participant for weaker elbow joint angles. The rest time between sets was 3 min. The total lifting weights of the dumbbell for 10 sessions were calculated for each subject in the EXT and FLE groups.

### MVC-ISO, MVC-CON, and MVC-ECC Torque

MVC-ISO torque was measured at 10° (MVC-ISO_10_), 50° (MVC-ISO_50_), 90° (MVC-ISO_90_), and 130° (MVC-ISO_130_) elbow flexion in 45° shoulder flexion, with the trunk and pelvis being secured with a belt, for the trained (dominant) and non-trained (non-dominant) arms using an isokinetic dynamometer (Biodex System 3.0, Biodex Medical Systems Inc., Shirley, NY, United States). The MVC-ISO torque measurements for each angle were performed in a random order, but the measurements of the trained (dominant) arm were taken before those of the non-trained (non-dominant) arm. Each contraction lasted for 3 s, and two measurements were made for each angle with a 45-s interval (Tseng et al., 2020), and the average of the two measures was used for further analysis. The average of MVC-ISO_10_, MVC-ISO_50_, MVC-ISO_90_, and MVC-ISO_130_ torque was also calculated as the average of the four angles (MVC-ISOave) torque and used for further analysis. During all measurements, verbal encouragement was provided to the participants.

The isokinetic dynamometer was also used to measure MVC-CON and MVC-ECC torque of the elbow flexors of the trained (dominant) and non-trained (non-dominant) arms in the same setting as that on MVC-ISO measures. MVC-CON torque was measured at 60°/s (MVC-CON_60_) and 180°/s (MVC-ISO_180_), and MVC-ECC torque was measured at 60°/s in this order. The rest time between measurements was 120 s, and the measurements for the trained (dominant) arm were performed before the non-dominant arm. The range of motion was 120° for the measurements, and the starting angle was 0° for MVC-CON, and 130° elbow flexion for MVC-ECC (Colson et al., 1999). MVC-CON and MVC-ECC torque was measured five times consecutively, and the maximum torque obtained was used for the subsequent analysis. The average of MVC-CON_60_ and MVC-ISO_180_ torque was also calculated as MVC-CON_*ave*_ torque and used for further analysis. During all measurements, the investigator gave verbal encouragement to the participants.

### Muscle Thickness

A total of biceps brachii and brachialis MT of the trained (dominant) and non-trained (non-dominant) arms was measured using B-mode ultrasonography with an 8-MHz linear probe (LOGIQ e V2; GE Healthcare Japan, Tokyo, Japan). The investigator minimized the pressure of the probe against the skin as much as possible, and the same investigator took all measurements at both baseline measurement (PRE) and post-training (POST). The measurement sites were 50% (MT_50_), 60%(MT_60_), and 70% (MT_70_) of the lateral epicondyle of the humerus from the acromion. Each participant lay in the supine position on a bed with the arms placed at each side and the forearm supinated while relaxing the arms. Ultrasound measurements of the transverse-axis were repeated twice, and the MT of biceps brachii plus brachialis was measured as the distance from the inner edge of the fascia to the humerus (Abe et al., 2000; Sato et al., 2021). The average of the two measurements was calculated for each site and used for further analysis. The average value (MT_*ave*_) of the MT at the 50, 60, and 70% sites was also calculated and used for further analysis.

### Test-Retest Reliability of the Measurement

The reliability of test and retest for the measured values of MVC-ISO torque, MVC-CON torque, MVC-ECC torque, and MT was assessed by the coefficient variation (CV) and the intraclass correlation coefficient (ICC) using 6 healthy men (25.0 ± 3.7 y, 168.0 ± 4.8 cm, 61.2 ± 4.4 kg) with 1 week between the two measures without any training. The CV of the measurements for MVC-ISO_10_, MVC-ISO_50_, MVC-ISO_90_, MVC-ISO_130_, MVC-ISO_*ave*_, MVC-CON_60_, MVC-CON_180_, MVC-ECC, and MT_*ave*_ were 6.1 2.6, 4.0 2.2, 3.5 2.5, 1.7 1.7, 2.3 0.6, 2.0 1.7, 4.7 2.7, 2.3 1.3, and 1.0 0.4%, respectively, and the ICC for the measurements were 0.85, 0.93, 0.89, 0.89, 0.96, 0.98, 0.84, 0.96, and 0.98, respectively.

### Statistical Analysis

Statistical analyses were performed using the SPSS version 24.0 (IBM Japan, Inc., Tokyo, Japan). The normality of the data was confirmed using a Shapiro-Wilk test. Group differences at the baseline were assessed using a one-way analysis of variance (ANOVA). A split-plot ANOVA with two factors [group (EXT vs. FLE vs. Control) × time (PRE vs. POST)] was used to compare between the groups for changes in MVC-ISO, MVC-CON, MVC-ECC torque, and MT in the trained arm and non-trained arm from pre- (PRE) to post-training (POST). Classification of effect size for the split-plot ANOVA results was based on η*p*^2^, and less than 0.01 was considered as a small, 0.02–0.1 was considered as a medium, and over 0.1 was considered as a large effect size (Cohen, 1988). A paired *t*-test with Bonferroni correction was used to determine significant differences between PRE and POST values when significant effects were found. Furthermore, when significant differences were found between PRE and POST values in the EXT and FLE groups, the magnitude of the change in each variable from PRE to POST was compared between the groups using a Man-Whitney *U*-test. The effect size (ES) was calculated as a difference in the mean values between pre- and post-training divided by the pooled SD (Cohen, 1988). ES of 0.00–0.19 was considered trivial, 0.20–0.49 was small, 0.50–0.79 was moderate, and ≥ 0.80 was large. In addition, an independent *t*-test was used to compare the total dumbbell weight lifted over the 10 sessions between the EXT and FLE groups. The differences were considered statistically significant at an alpha level of 0.05. Descriptive data are shown as mean ± standard deviations (SD).

## Results

### Training

All participants in both training groups completed all training sessions as planned. The total dumbbell weight lifted in the 10 training sessions was 3,033 ± 617 kg in the EXT group and 4,251 ± 1,515 kg in the FLE group, and the total weight was lower (*p* = 0.02) for the EXT than FLE group. However, when the total dumbbell weight lifted was normalized by the baseline MVC-ISO_50_ or MVC-ISO_90_ torque (elbow flexor torque at the starting elbow joint angle) converted to “kg” using the acceleration of gravity (9.8) and forearm length, no significant difference (*p* = 0.43) between the EXT (212.0 ± 2.2) and FLE (213.6 ± 3.4) was evident.

### MVC-ISO, MVC-CON, and MVC-ECC Torque

Changes in MVC-ISO, MVC-CON, and MVC-ECC torque of the trained and non-trained arms from pre- to post-training are shown in Table 1. For the trained arm, significant interaction effects were evident for MVC-ISO_50_ (*p* = 0.006), MVC-ISO_90_ (*p* = 0.031), MVC-CON_60_ (*p* = 0.009), MVC-CON_180_ (*p* = 0.041), and MVC-ECC (*p* = 0.03) torque, but not for MVC-ISO_10_ and MVC-ISO_130_ torque. A significant time effect was found for all torque measures except for MVC-ISO_90_ and MVC-ISO_130_ torque. The *post hoc* test showed that MVC-ISO_50_ (*p* = 0.014, *d* = 0.58), MVC-ISO_90_ (*p* = 0.03, *d* = 0.51), MVC-CON_60_ (*p* = 0.02, *d* = 0.65), MVC-CON_180_ (*p* = 0.019, *d* = 0.57), and MVC-ECC torque (*p* < 0.01, *d* = 0.60) increased only after EXT. No significant changes in all variables were observed for the control group.

**TABLE 1:** Changes (mean ± SD) in maximum voluntary isometric contraction (MVC-ISO) torque at four different angles (10°: MVC-ISO_10_, 50°: MVC-ISO_50_, 90°: MVC-ISO_90_, and 130°: MVC-ISO_130_), concentric contraction (MVC-CON) at two different velocities (60°/s: MVC-CON_60_, 180°/s: MVC-CON_180_), eccentric contraction (MVC-ECC), and muscle thickness (biceps brachii plus brachialis) at the 50% (MT_50_), 60% (MT_60_), and 70%(MT_70_) of the proximal-distal distance of the upper arm of the trained and non-trained arms before (PRE) and after (POST) 5-weeks training at more extended elbow joint angles (EXT) or more flexed elbow joint angles (FLE), and control group without training.

		Trained arm			Non-trained arm		
			
Variables	Group	Pre	Post	ANOVA results, partial η ^2^ (η *_*p*_*^2^)	Pre	Post	ANOVA results, partial η ^2^ (η *_*p*_*^2^)
MVC-ISO_10_ (Nm)	EXT	25.0 ± 6.61	27.9 ± 6.5	**T:***F* = 13.10, η*_*p*_*^2^ = 0.31	24.2 ± 5.2	25.8 ± 6.2	**T:***F* = 2.26, η*_*p*_*^2^ = 0.07
	FLE	29.0 ± 9.1	31.7 ± 10.4	**G × T:***F* = 0.86, η*_*p*_*^2^ = 0.06	26.8 ± 11.0	28.7 ± 11.7	**G × T:***F* = 0.29, η*_*p*_*^2^ = 0.02
	Control	23.4 ± 5.5	24.4 ± 6.5		21.4 ± 8.1	21.7 ± 7.3	
MVC-ISO_50_ (Nm)	EXT	35.5 ± 8.6	40.5 ± 9.0*	**T:***F* = 4.52, η*_*p*_*^2^ = 0.14	32.1 ± 9.3	36.1 ± 8.6*	**T:***F* = 1.17, η*_*p*_*^2^ = 0.04
	FLE	42.3 ± 13.9	43.2 ± 14.3	**G × T:***F* = 6.23, η*_*p*_*^2^ = 0.31	39.6 ± 17.0	39.9 ± 15.4	**G × T:***F* = 4.28, η*_*p*_*^2^ = 0.23
	Control	38.7 ± 11.7	37.4 ± 12.0		35.0 ± 12.9	33.3 ± 11.5	
MVC-ISO_90_ (Nm)	EXT	37.7 ± 9.2	42.6 ± 9.8*	**T:***F* = 1.09, η*_*p*_*^2^ = 0.04	33.8 ± 9.7	39.1 ± 8.6*	**T:***F* = 3.75, η*_*p*_*^2^ = 0.11
	FLE	47.4 ± 17.6	49.0 ± 16.6	**G × T:***F* = 3.93, η*_*p*_*^2^ = 0.21	42.4 ± 14.8	41.8 ± 14.6	**G × T:***F* = 6.69, η*_*p*_*^2^ = 0.32
	Control	45.6 ± 16.6	42.6 ± 14.1		39.5 ± 12.4	39.3 ± 11.3	
MVC-ISO_130_ (Nm)	EXT	27.4 ± 8.0	33.3 ± 10.4	**T:***F* = 3.28, η*_*p*_*^2^ = 0.11	25.1 ± 7.9	30.1 ± 9.6	**T:***F* = 1.90, η*_*p*_*^2^ = 0.06
	FLE	33.5 ± 14.5	35.5 ± 14.6	**G × T:***F* = 1.87, η*_*p*_*^2^ = 0.12	31.4 ± 12.5	31.0 ± 13.6	**G × T:***F* = 3.13, η*_*p*_*^2^ = 0.18
	Control	38.8 ± 14.5	38.2 ± 13.4		34.0 ± 14.4	33.8 ± 11.0	
MVC-CON_60_ (Nm)	EXT	30.3 ± 8.4	35.4 ± 7.3*	**T:***F* = 6.99, η*_*p*_*^2^ = 0.19	28.2 ± 6.0	33.1 ± 6.3*	**T:***F* = 4.74, η*_*p*_*^2^ = 0.14
	FLE	33.1 ± 12.7	36.3 ± 11.7	**G × T:***F* = 5.54, η*_*p*_*^2^ = 0.28	31.7 ± 12.2	32.4 ± 12.9	**G × T:***F* = 5.03, η*_*p*_*^2^ = 0.26
	Control	32.7 ± 7.9	30.9 ± 9.0		29.9 ± 9.4	29.2 ± 8.8	
MVC-CON_180_ (Nm)	EXT	23.0 ± 7.2	27.0 ± 7.0*	**T:***F* = 6.56, η*_*p*_*^2^ = 0.19	22.5 ± 6.7	25.0 ± 7.1	**T:***F* = 7.57, η*_*p*_*^2^ = 0.21
	FLE	26.1 ± 8.7	26.6 ± 8.7	**G × T:***F* = 3.57, η*_*p*_*^2^ = 0.20	23.9 ± 9.6	24.8 ± 9.5	**G × T:***F* = 1.89, η*_*p*_*^2^ = 0.12
	Control	21.7 ± 5.8	22.2 ± 6.3		20.4 ± 5.3	20.8 ± 6.3	
MVC-ECC (Nm)	EXT	41.8 ± 12.1	48.4 ± 10.1*	**T:***F* = 11.5, η*_*p*_*^2^ = 0.28	39.0 ± 10.7	43.1 ± 9.4*	**T:***F* = 9.07, η*_*p*_*^2^ = 0.24
	FLE	45.1 ± 15.8	46.1 ± 15.6	**G × T:***F* = 3.99, η*_*p*_*^2^ = 0.22	41.8 ± 15.4	42.4 ± 16.1	**G × T:***F* = 2.55, η*_*p*_*^2^ = 0.15
	Control	48.0 ± 14.8	49.8 ± 14.5		45.2 ± 13.4	46.6 ± 12.9	
MT_50_ (mm)	EXT	21.8 ± 4.4	22.9 ± 4.2*	**T:***F* = 9.70, η*_*p*_*^2^ = 0.25	20.6 ± 4.0	20.4 ± 4.9	**T:***F* = 0.10, η*_*p*_*^2^ < 0.01
	FLE	21.9 ± 5.1	22.7 ± 5.1*	**G × T:***F* = 6.91, η*_*p*_*^2^ = 0.32	20.5 ± 5.1	20.5 ± 4.8	**G × T:***F* = 0.92, η*_*p*_*^2^ = 0.06
	Control	21.5 ± 4.2	21.1 ± 4.7		20.1 ± 4.0	20.2 ± 3.6	
MT_60_ (mm)	EXT	21.8 ± 4.1	23.3 ± 4.0*	**T:***F* = 25.4, η*_*p*_*^2^ = 0.47	20.5 ± 4.6	20.6 ± 4.6	**T:***F* = 2.33, η*_*p*_*^2^ = 0.07
	FLE	21.9 ± 5.3	23.0 ± 5.6*	**G × T:***F* = 5.93, η*_*p*_*^2^ = 0.29	19.9 ± 4.9	20.2 ± 4.8	**G × T:***F* = 0.47, η*_*p*_*^2^ = 0.03
	Control	21.3 ± 4.5	21.4 ± 4.7		20.3 ± 3.5	20.7 ± 3.7	
MT_70_ (mm)	EXT	23.7 ± 3.7	26.7 ± 3.6*	**T:***F* = 55.5, η*_*p*_*^2^ = 0.66	21.9 ± 4.1	22.2 ± 4.1	**T:***F* = 7.71, η*_*p*_*^2^ = 0.21
	FLE	23.5 ± 5.0	24.0 ± 5.0	**G × T:***F* = 27.7, η*_*p*_*^2^ = 0.66	21.4 ± 5.0	21.6 ± 5.3	**G × T:***F* = 0.78, η*_*p*_*^2^ = 0.02
	Control	22.8 ± 4.6	23.2 ± 4.4		21.8 ± 4.0	22.2 ± 3.8	

*The two-way ANOVA results (T: time effect, G* **×** T: group × time interaction effect; F-value) and partial η^2^ (η_*p*_^2^) are shown for the trained and non-trained arms. *: significant (p < 0.05) difference from the PRE value.

For the non-trained arm, significant interaction effects were evident for MVC-ISO_50_ (*p* = 0.023), MVC-ISO_90_ (*p* = 0.004), and MVC-CON_60_ (*p* = 0.013) torque, and a significant time effect was found for MVC-CON_60_, MVC-CON_180_, MVC-ECC torque. The *post hoc* test showed that MVC-ISO_50_ (*p* = 0.017, *d* = 0.44), MVC-ISO_90_ (*p* = 0.012, *d* = 0.58), and MVC-CON_60_ torque (*p* = 0.029, *d* = 0.79) increased only after EXT. The FLE and control groups did not show any significant changes in the variables.

**FIGURE 1:**
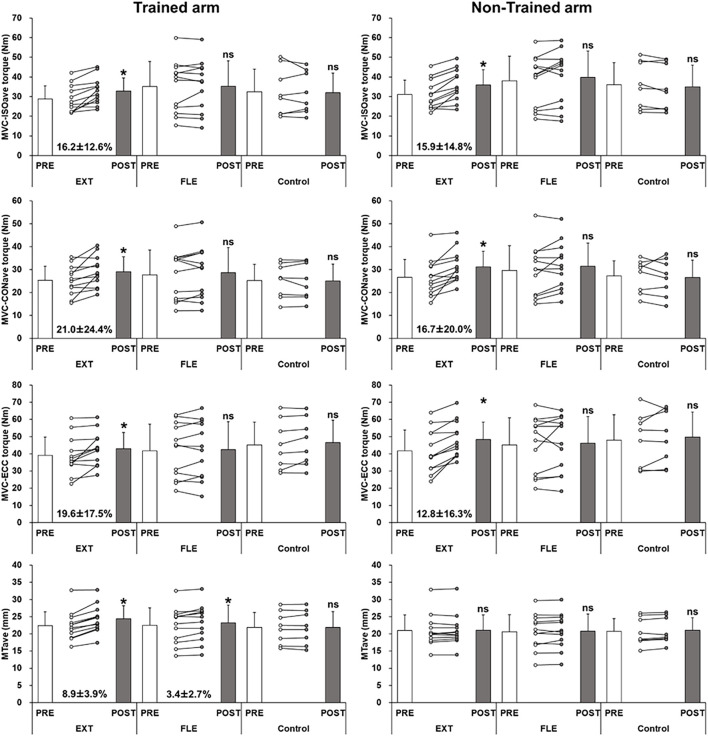
Changes (mean ± SD, individuals) in average maximum voluntary isometric contraction torque of four different angles (MVC-ISO_*ave*_), concentric contraction torque at two different velocities (MVC-CON_*ave*_), eccentric contraction (MVC-ECC) torque, and muscle thickness at three positions (MT_*ave*_) before (PRE) and after (POST) 5-week training at more extended elbow joint angles (EXT) or more flexed elbow joint angles (FLE), and for the control group without training in trained arm. *: significant (*p* < 0.05) difference from the PRE-value, ns: no significant difference from the PRE-value.

Figure 1 shows changes in MVC-ISO_*ave*_, MVC-CON_*ave*_ and MVC-ECC torque of individual participants and the group mean (± SD) values for the trained and non-trained arms. For the trained arm, the *post hoc* test showed that MVC-ISO_*ave*_ (*p* < 0.01, *d* = 0.64), MVC-CON_*ave*_ (*p* = 0.010, *d* = 0.62) and MVC-ECC torque (*p* < 0.01, *d* = 0.60) increased only after EXT. In addition, for the non-trained arm, the *post hoc* test showed that MVC-ISO_*ave*_ (*p* < 0.01, *d* = 0.62), and MVC-CON_*ave*_ torque (*p* = 0.028, *d* = 0.58) increased only after EXT.

### Biceps Brachii Plus Brachialis MT

Changes in MT of biceps brachii plus brachialis in the trained and non-trained arms from pre- to post-training are shown in Table 1 and Figure 1. For the trained arm, significant interaction, as well as time effect, was evident for all measures. The *post hoc* test showed that MT_50_ and MT_60_ increased (*p* < 0.05) similarly after EXT and FLE, but MT_70_ increased (*p* < 0.01, *d* = 0.82) only after EXT. The increase in MT_*ave*_ was greater (*p* < 0.01) for the EXT (8.9 ± 3.9%) than FLE group (3.4 ± 2.7%). For the non-trained arm, no significant interaction effects in all MT variables, and a significant time effect was found for only MT_70_. The control group did not show any changes in MT for both arms.

## Discussion

We tested the hypotheses; that (1) the increases in muscle strength and muscle thickness of the trained arm would be greater for the EXT than FLE group, and (2) the increases in muscle strength of the non-trained arm (i.e., cross-education effect) would be also greater for the EXT than FLE group. The results showed that (1) MVC-ISO_50_, MVC-ISO_90_, MVC-ISO_*ave*_, MVC-CON_60_, MVC-CON_180_, MVC-CON_*ave*_, and MVC-ECC torque of the trained arm increased significantly only for the EXT group; (2) muscle thickness (MT) of the trained arm increased greater for the EXT than FLE group; and (3) MVC-ISO_50_, MVC-ISO_90_, MVC-ISO_*ave*_, MVC-CON_60_, and MVC-CON_*ave*_ torque of the non-trained arm increased significantly only for the EXT group. These results were in line with the hypotheses.

The magnitude of the change in MVC-ISO_90_ torque of the trained arm after 10 training sessions in the EXT group was 14.1 ± 17.1% (Table 1). Tseng et al. (2020) reported 19% increase in MVC-ISO_90_ torque in the trained arm after eccentric-only training of the elbow flexors performed once a week for 5 weeks with a gradual increase in the intensity from 10 to 100% of MVC-ISO_90_ torque. This protocol was similar to that of the present study, but the number of sessions per week in the present study was doubled. When comparing to the increase found by Tseng et al. (2020), the magnitude of the increases in the MVC-ISO_90_ torque in the present study was smaller. The greater increase in the strength in the study by Tseng et al. (2020) may be due to the focus on eccentric contractions. Valdes et al. (2021) reported that elbow flexor eccentric-only resistance training performed by the dominant (non-immobilized) arm 3 times a week with 80–120% of one concentric 1-RM load increased MVC-ISO strength (20.9%) greater than concentric-eccentric coupled resistance training (13.7%) in which 60–90% of 1-RM load was used for the same total training volume. It seems possible that eccentric-only resistance training is more effective for increasing muscle strength. It is interesting to compare the eccentric-only resistance training and conventional resistance training consisting of both eccentric and concentric contractions performed at long vs. short muscle length conditions for changes in MVC-ISO strength.

In the trained arm, significant increases in MVC-ISO_50_, MVC-ISO_90_, MVC-ISO_*ave*_, MVC-CON_60_, MVC-CON_180_, MVC-CON_*ave*_, and MVC-ECC torque were found only after EXT, indicating that the EXT was more effective than FLE for increasing muscle strength. It is important to note that the normalized total weight and time under the tension were the same between the EXT and FLE groups; thus the different training effects on the muscle strength were most likely due to the difference in the elbow joint angles in training, presumably a difference in muscle lengths. The exact difference in muscle lengths between EXT and FLE protocols was not known, but it was assumed that the biceps brachii and brachialis muscle lengths were longer in the EXT than FLE. Nosaka et al. (2005) compared maximal eccentric contractions of the elbow flexors from 50° flexion to a full extension (0°) and those from 130° to 80° flexion and found greater muscle damage for the former than the latter. They speculated that eccentric contractions at long muscle lengths induced greater muscle damage than those at short muscle lengths. The present study also used the same elbow joint ROM setting for the two conditions to those of the study by Nosaka et al. (2005), although the movements included both concentric and eccentric contractions in the present study. It seems likely that the EXT was performed at longer muscle lengths than the FLE, but this has to be confirmed in a future study.

The greater training effects by long than short muscle length resistance training have been reported. For example, Noorkõiv et al. (2015) compared long (knee joint angle: 87.5 ± 6.0°) and short (38.1 ± 3.7°) muscle length isometric training of the knee extensors consisting of 5 sets of 5-s maximal contractions performed three times a week for 6 weeks. They reported a significant increase in peak isokinetic concentric torque at 30°/s (13%) and 120°/s (12%) only for the long muscle length group. They also showed that only the long muscle length isometric contraction training increased maximal voluntary concentric contraction strength (Noorkõiv et al., 2015). McMahon et al. (2014) compared changes in isometric strength of the knee extensors at 70° of knee flexion between long muscle length (40–90° knee flexion) and short muscle length (0–50°) training protocols consisting of concentric and eccentric contractions at 55% 1-RM for the long muscle length condition and 80% 1-RM for the short muscle length condition performed three times a week for 8 weeks. They showed a significantly greater increase in MVC-ISO strength of the knee extensors for the long (26%) than short muscle length protocol (7%). They speculated that the long muscle length protocol imposed greater activation and metabolic demand in producing force than the short muscle length protocol, which might contribute to the greater strength increase after the long than short protocol (McMahon et al., 2014). Indeed, Carr et al. (2021) reported a greater reduction in maximal force due to fatigue after intermittent maximal isometric contractions at a long than a short muscle length in biceps brachii. This may also be the case for the present study, but the differences between the EXT and FLE protocols for muscle activity and mechanical stimuli are not known. As discussed below, it is also possible that the greater muscle hypertrophy induced by the EXT than FLE contributed to the greater increases in muscle strength after EXT. However, it should be noted that MVC-ISO_10_ and MVC-ISO_130_ did not show significant interaction effects (Table 1). This may be attributed to the low training volume in the present study, in which the training intensity was 10–30% of MVC in the first three out of 10 sessions. It may be that all muscle strength measures would have increased if more training sessions were added after the 10th session to increase the training volume.

Regarding the MT, the magnitude of increase in MT_*ave*_ of the trained arm in the EXT group (8.6%) was greater than that of the FLE group (3.3%). It should be noted that a greater increase in MT was observed at the distal region of muscle (MT_70_: 12.8%) than the proximal region (MT_50_: 5.4%, MT_60_: 7.1%) in the EXT group (Table 1). Muscle hypertrophy occurs in homogeneously between different regions in a muscle, and resistance training mode also influences the region-specific hypertrophy (Antonio, 2000; Franchi et al., 2014; Diniz et al., 2020). For example, Franchi et al. (2014) compared the changes in CSA of vastus lateralis muscle at different regions using MRI along after eccentric-only vs. concentric-only training of the knee extensors with 80% 1-RM load performed three times a week for 10 weeks. They reported that eccentric-only training and concentric-only training increased CSA in the mid-portion of the vastus lateralis by 7 and 11%, respectively, and in the distal portion by 8 and 2%, respectively, suggesting that muscle hypertrophy in the distal portion was greater after eccentric-only than concentric-only training. It may be that the training at longer muscle lengths produced greater brachialis hypertrophy due to greater mechanical stimuli generated especially in the long muscles in eccentric contractions.

In the non-trained arm, MVC-ISO_50_, MVC-ISO_90_, MVC-ISO_*ave*_, MVC-CON_60_, and MVC-CON_*ave*_ torque increased significantly only after EXT, indicating a greater cross-education effect for the EXT. The lack of increase in the other muscle strength measures was probably due to the low intensity of training in the first half of the training period (1–3 sessions) and the lower training volume, as mentioned above. The factors that influence the magnitude of the cross-education effect include training intensity (Colomer-Poveda et al., 2020), muscle contraction type (Hortobagyi et al., 1997; Kidgell et al., 2015; Manca et al., 2017; Tseng et al., 2020; Valdes et al., 2021), number of sessions (Barss et al., 2018), and intervention duration (Manca et al., 2021). Some studies have reported that the cross-education effect is greater after eccentric than concentric resistance training (Hortobagyi et al., 1997; Kidgell et al., 2015; Tseng et al., 2020) or concentric-eccentric coupled training (Valdes et al., 2021). This may be attributed to the greater central nervous system adaptations, such as the reduction of intracortical and interhemispheric inhibition after eccentric than concentric resistance training (Kidgell et al., 2015).

Kidgell et al. (2015) reported that 4 weeks of maximal eccentric training of the wrist flexors resulted in greater reductions in both ipsilateral intracortical inhibition (32%) and silent period duration (15–27%) when compared with maximal concentric resistance training (2 and 4–8%, respectively). Moreover, Tseng et al. (2020) showed that MVC-ISO_90_ torque in the non-trained arm increased by 11 and 5% for eccentric-only and concentric-only training, respectively, indicating superiority of eccentric to concentric training. In the present study, MVC-ISO_90_ torque of the non-trained arm was increased 13% after EXT, which was similar to that reported by Tseng et al. (2020). It may be that muscle length during resistance training is also a factor affecting the cross-education effect. It is interesting to investigate whether eccentric-only training at longer muscle lengths produces a greater cross-education effect than coupled concentric-eccentric training or concentric-only training. Regarding MT, no cross-education effect was observed after either EXT or FLE in the present study. Previous studies also reported no significant muscle hypertrophy for the non-trained arm after unilateral resistance training (Kidgell et al., 2015; Tseng et al., 2020). Thus, the cross-education effect on muscle hypertrophy does not appear to exist.

The present study had several limitations. Firstly, the present study did not assess actual muscle length changes in EXT and FLE protocols. Thus the exact difference in the muscle length for its training effects, including the cross-education effect, was not known. Secondly, the intervention period (5 weeks) was short. A longer intervention period could have better clarified the adaptations in the trained and non-trained arms by EXT and FLE. Thirdly, MT was used as a parameter of muscle hypertrophy in the present study. Future studies should use magnetic resonance imaging which is the gold standard for measuring muscle volume. Fourthly, the present study used untrained young, healthy adults as participants. It is not known whether the results of the present study are applicable to trained individuals, older adults, and clinical populations. Lastly, neurophysiological measures such as electromyography and transcranial magnetic stimulation were not included in the present study. Future studies should examine the difference in the adaptation of the nervous system between EXT and FLE. With these limitations, the findings of the present study appear to show possible greater effects of long muscle length resistance training in rehabilitation and athletic training.

## Conclusion

In conclusion, the 5-week unilateral progressive resistance training performed at more extended elbow joint (EXT) induced greater increases in muscle strength of trained arm and cross-education effect on the non-trained arm than that at flexed elbow joint angle (FLE), and an increase in muscle thickness of the trained arm was greater after EXT than FLE with the same time under tension. These results suggest that the EXT resistance exercise training is more effective than the FLE with the same ROM for the elbow flexors to increase both trained and non-trained contralateral arms muscle strength and muscle size in the trained arm. It seems likely that muscle length in resistance exercise training is an important factor for its outcomes. It is interesting to investigate if this is also the case for other muscles.

## Data Availability Statement

The raw data supporting the conclusions of this article will be made available by the authors, without undue reservation.

## Ethics Statement

This study was approved by the Ethics Committee of the Niigata University of Health and Welfare, Niigata, Japan (Procedure #18442). The patients/participants provided their written informed consent to participate in this study.

## Author Contributions

SS, KN, and MN designed the study, drafted, and revised the manuscript. SS, RY, RK, KaY, KoY, JN, and MN contributed to the data collection and analyses. JN made critical revisions to the manuscript. All authors approved the final version of the manuscript.

## Conflict of Interest

The authors declare that the research was conducted in the absence of any commercial or financial relationships that could be construed as a potential conflict of interest.

## Publisher’s Note

All claims expressed in this article are solely those of the authors and do not necessarily represent those of their affiliated organizations, or those of the publisher, the editors and the reviewers. Any product that may be evaluated in this article, or claim that may be made by its manufacturer, is not guaranteed or endorsed by the publisher.
